# Supplementary material for: Auxin Metabolome Profiling in the Arabidopsis Endoplasmic Reticulum Using an Optimised Organelle Isolation Protocol
Source: Int J Mol Sci. 2021 Aug 29;22(17):9370. doi: 10.3390/ijms22179370 (PMC8431077; doi:10.3390/ijms22179370)
Supplement: Supplementary file 1 [file ijms-22-09370-s001.zip › ijms-1343826-supplementary.pdf]

International Journal of *Molecular Sciences*

MDPI

Supplementary materials

Auxin Metabolome Profiling in the Arabidopsis Endoplasmic Reticu-lum Using an Optimised Organelle Isolation Protocol

Ludmila Včelařová †, Vladimír Skalický †, Ivo Chamrád, René Lenobel, Martin F. Kubeš ‡, Aleš Pěnčík * and Ondřej Novák

Laboratory of Growth Regulators, Institute of Experimental Botany of the Czech Academy of Sciences, and Faculty of Science, Palacký University, Šlechtitelů 27, CZ-78371 Olomouc, Czech Republic; ludmila.vcelarova@upol.cz (L.V.); vladimir.skalicky@upol.cz (V.S.); ivo.chamrad@upol.cz (I.C.); rene.lenobel@upol.cz (R.L.); martin.kubes@uhk.cz (M.F.K.); ondrej.novak@upol.cz (O.N.).
* Correspondence: ales.pencik@upol.cz; Tel.: +420-585-634-853
† These authors contributed equally to this work.
‡ Current address: Department of Biology, Faculty of Science, University of Hradec Králové, Rokitanského 62, 500 03 Hradec Králové, Czech Republic.

Citation: Včelařová, L.; Skalický, V.; Chamrád, I.; Lenobel, R.; Kubeš, M.F.; Pěnčík, A.; Novák, O. Auxin Metabolome Profiling in the Arabidopsis Endoplasmic Reticulum Using an Optimised Organelle Isolation Protocol. *Int. J. Mol. Sci.* **2021**, *22*, 9370. https://doi.org/10.3390/ijms22179370

Academic Editor: Jutta Ludwig-Mueller

Received: 31 July 2021
Accepted: 25 August 2021
Published: 29 August 2021

Publisher's Note: MDPI stays neutral with regard to jurisdictional claims in published maps and institutional affiliations.

Copyright: © 2021 by the authors. Submitted for possible open access publication under the terms and conditions of the Creative Commons Attribution (CC BY) license (http://creativecommons.org/licenses/by/4.0/).

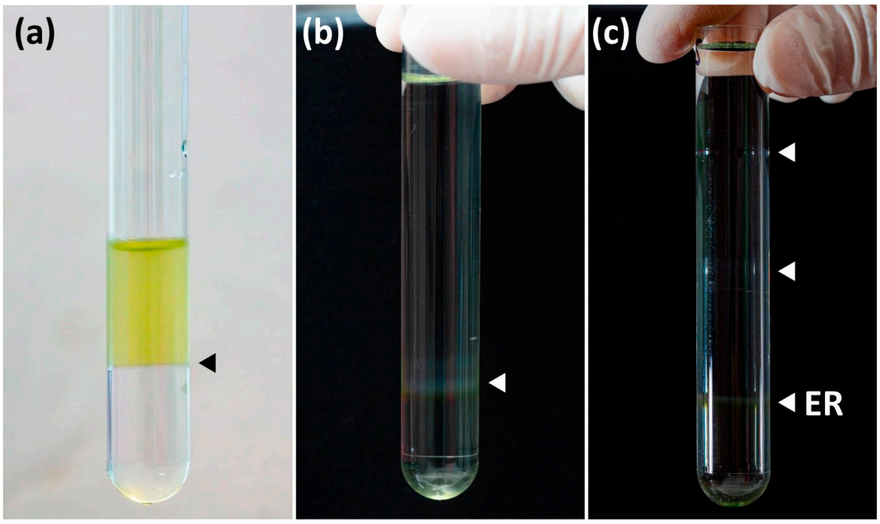

Figure S1. Procedure for isolating the ER-enriched fraction by density gradient ultracentrifugation. (**a**) A supernatant S4,000 loaded on the top of 31% (w/w) sucrose solution. (**b**) A two-step density gradient after ultracentrifugation. The supernatant was removed and focused microsomes were overlaid with 27%, 19%, and 8% (w/w) sucrose solutions. (**c**) A four-step gradient after the final ultracentrifugation. The 27/31% interface enriched with ER microsomes was collected. Arrows indicate interphases between the two gradient-forming solutions, ER – endoplasmic reticulum-enriched fraction.

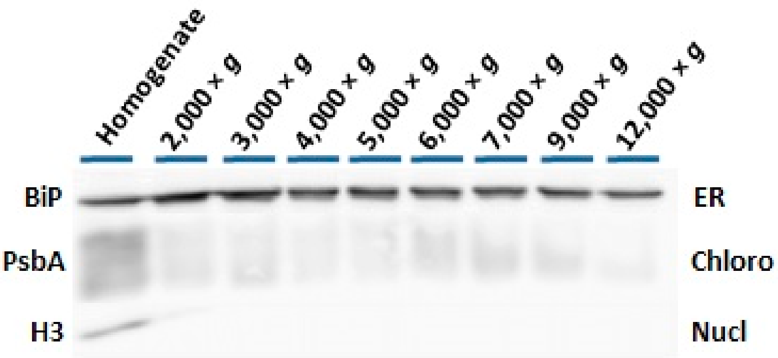

Figure S2. Elimination of co-migrating chloroplasts by optimizing the initial centrifugation prior to density-gradient separation. The crude homogenate was centrifuged at speeds between 2,000 and 12,000 × *g*. The parent homogenate and the resulting supernatants were then subjected to Western blot analysis. The following organelle-specific markers were immunodetected: endoplasmic reticulum – ER (Lumena-binding protein, BiP), chloroplasts – Chloro (D1 protein of photosystem II, PsbA) and nuclei – Nucl (Histone 3, H3).

Isolation					
Replicate 1	Replicate 1				
Replicate 2	74.40	Replicate 2			
Replicate 3	76.40	76.80	Replicate 3		
Replicate 4	76.70	74.00	73.00	Replicate 4	
Replicate 5	68.20	67.00	71.90	69.70	Replicate 5

Figure S3. Reproducibility of ER isolations visualized using a histogram showing pairwise comparisons of protein identifications (in %).

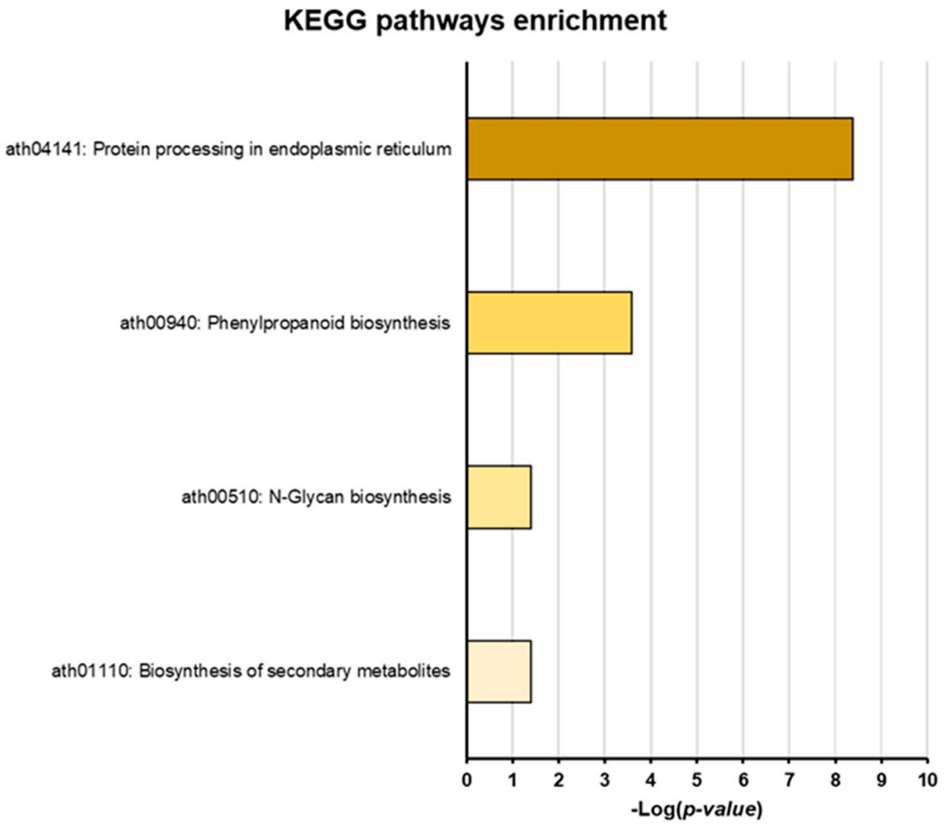

Figure S4. KEGG pathway enrichment analysis. Significantly enriched KEGG pathways are sorted according to the –Log of their p-value.

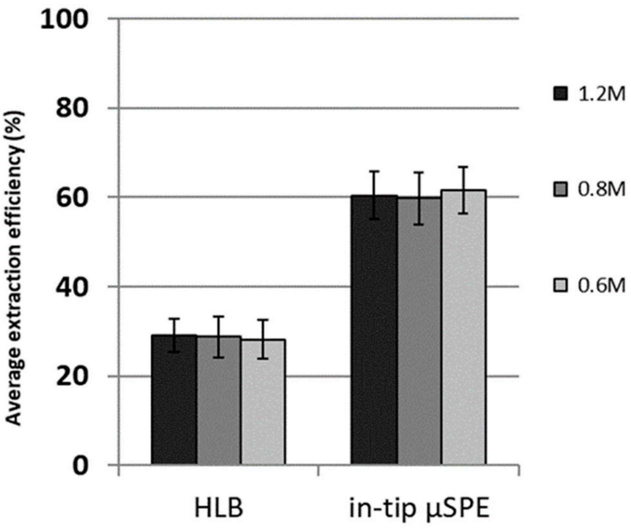

Figure S5. Total process efficiency (%) of solid-phase extraction (SPE) protocols. The recovery achieved using a conventional HLB column [1] was compared to that for an in-tip µSPE protocol [2]. Gradient-forming sucrose solutions were spiked with a mixture of six auxin standards (1 pmol each). Recoveries of standards were evaluated by LC-MS/MS. Values are mean recoveries (%) of all tested auxin standards (n=3). Error bars indicate the s. d.

Tables S1–S4. Tables are available in attached in zip file.

Table S1. Protein and peptide identification characteristics for analyses of ER isolates and control samples (n=5 for both) as provided by MaxQuant software version 1.6.10.43 [3].

Table S2. A list of all identified proteins assigned as ER-located.

Table S3. Complete functional annotation clustering results for the proteins assigned as ER-located, as downloaded from DAVID Bioinformatics Resources 6.8 [4].

Table S4. Complete results of a KEGG pathways enrichment analysis for the proteins assigned as ER-located as downloaded from DAVID Bioinformatics Resources 6.8 [4].

Table S5. Levels of IAA metabolites levels and their relative abundance in Arabidopsis seedlings, an organelle suspension, and the ER-enriched fraction. Values are mean (n=5).

**Compound**	**Crude extract**		**Organelle suspension**		**ER fraction**	
	(pmol/g FW)	*(%)*	*(fmol/ml)*	*(%)*	*(fmol/ml)*	*(%)*
IAA	102.1	1.7	11,451.4	4.8	832.3	8.8
IAAsp	25.1	0.4	2,265.7	1.0	15.5	0.2
IAGlu	100.3	1.7	9,835.0	4.1	111.0	1.2
IAA-glc	78.2	1.3	8,883.5	3.7	118.3	1.2
oxIAA	909.2	15.6	36,105.3	15.2	975.6	10.3
oxIAA-glc	4,629.0	79.2	168,948.3	71.1	7,455.4	78.4

Table S6. Endogenous levels (fmol/µg of proteins) of auxin metabolites in the Arabidopsis crude extract and ER-enriched fraction. The mean abundance ± s.d. is given (n=5) for indole-3-acetic acid (IAA), IAA-aspartate (IAAsp), IAA-glutamate (IAGlu), IAA-glucose (IAA-glc), 2-oxoindole-3-acetic acid (oxIAA), oxIAA-glucose (oxIAA-glc)

**Compound**	**Crude extract**	**ER fraction**
	fmol/µg of proteins	
IAA	11.7 ± 0.9	718.1 ± 34.0
IAAsp	2.9 ± 0.1	13.3 ± 2.7
IAGlu	11.5 ± 0.9	95.7 ± 18.3
IAA-glc	8.9 ± 0.8	102.0 ± 16.6
oxIAA	103.9 ± 10.8	841.7 ± 150.0
oxIAA-glc	529.0 ± 13.7	6,432.1 ± 1,244.9

References

1. Novák, O.; Hényková, E.; Sairanen, I.; Kowalczyk, M.; Pospíšil, T.; Ljung, K. Tissue-specific profiling of the Arabidopsis thaliana auxin metabolome. *Plant J.* **2012**, *72*, 523–36, doi:10.1111/j.1365-313X.2012.05085.x.
2. Pěnčík, A.; Casanova-Sáez, R.; Pilařová, V.; Žukauskaitė, A.; Pinto, R.; Luis Micol, J.; Ljung, K.; Novák, O. Ultra-rapid auxin metabolite profiling for high-throughput mutant screening in Arabidopsis. *J. Exp. Bot.* **2018**, *69*, 2569–2579, doi:10.1093/jxb/ery084.

3. Tyanova, S.; Temu, T.; Cox, J. The MaxQuant computational platform for mass spectrometry-based shotgun proteomics. *Nat. Protoc.* **2016**, *11*, 2301–2319, doi:10.1038/nprot.2016.136.
4. Huang, D. W.; Sherman, B. T.; Lempicki, R. A. Systematic and integrative analysis of large gene lists using DAVID bioinformatics resources. *Nat. Protoc.* **2009**, *4*, 44–57, doi:10.1038/nprot.2008.211.
